# Comorbidity of self-harm and disordered eating in young people: evidence from a UK population-based cohort

**DOI:** 10.1192/bjo.2021.84

**Published:** 2021-06-18

**Authors:** Helen Bould, Naomi Warne, Jon Heron, Becky Mars, Paul Moran, Anne Stewart, Marcus Munafo, Lucy Biddle, Andy Skinner, David Gunnell

**Affiliations:** 1 Centre for Academic Mental Health, Population Health Sciences, Bristol Medical School, University of Bristol, Gloucestershire Health and Care NHS Foundation Trust, MRC Integrative Epidemiology Unit, University of Bristol Medical School; 2 Centre for Academic Mental Health, Population Health Sciences, Bristol Medical School, University of Bristol; 3 Department of Psychiatry, University of Oxford; 4 School of Psychological Science, University of Bristol, MRC Integrative Epidemiology Unit, University of Bristol Medical School, NIHR Biomedical Research Centre, University Hospitals Bristol NHS Foundation Trust; 5 School of Psychological Science, University of Bristol, MRC Integrative Epidemiology Unit, University of Bristol Medical School

## Abstract

**Aims:**

Self-harm and eating disorders are often comorbid in clinical samples but their co-occurrence in the general population is unclear. Given that only a small proportion of individuals who self-harm or have disordered eating present to clinical services, and that both self-harm and eating disorders are associated with substantial morbidity and mortality, we aimed to study these behaviours at a population level.

**Method:**

We assessed the co-occurrence of self-harm and disordered eating behaviours in 3384 females and 2326 males from a UK population-based cohort: the Avon Longitudinal Study of Parents and Children (ALSPAC). Participants reported on their self-harm and disordered eating behaviours (fasting, purging, binge-eating and excessive exercise) in the last year via questionnaire at 16 and 24 years. At each age we assessed how many individuals who self-harm also reported disordered eating, and how many individuals with disordered eating also reported self-harm.

**Result:**

We found high comorbidity of self-harm and disordered eating. Almost two-thirds of 16-year-old females, and two-in-five 24-year-old males who self-harmed also reported some form of disordered eating. Young people with disordered eating reported higher levels of self-harm at both ages compared to those without disordered eating.

**Conclusion:**

As self-harm and disordered eating commonly co-occur in young people in the general population, it is important to screen for both sets of difficulties to provide appropriate treatment.

